# Supplementary material for: ‘ZOOMing’ in on Consulting with Children and Parents Remotely to Co-Create Health Information Resources
Source: Children (Basel). 2023 Mar 11;10(3):539. doi: 10.3390/children10030539 (PMC10047904; doi:10.3390/children10030539)
Supplement: Supplementary file 1 [file children-10-00539-s001.zip › children-2246923-supplementary.pdf]

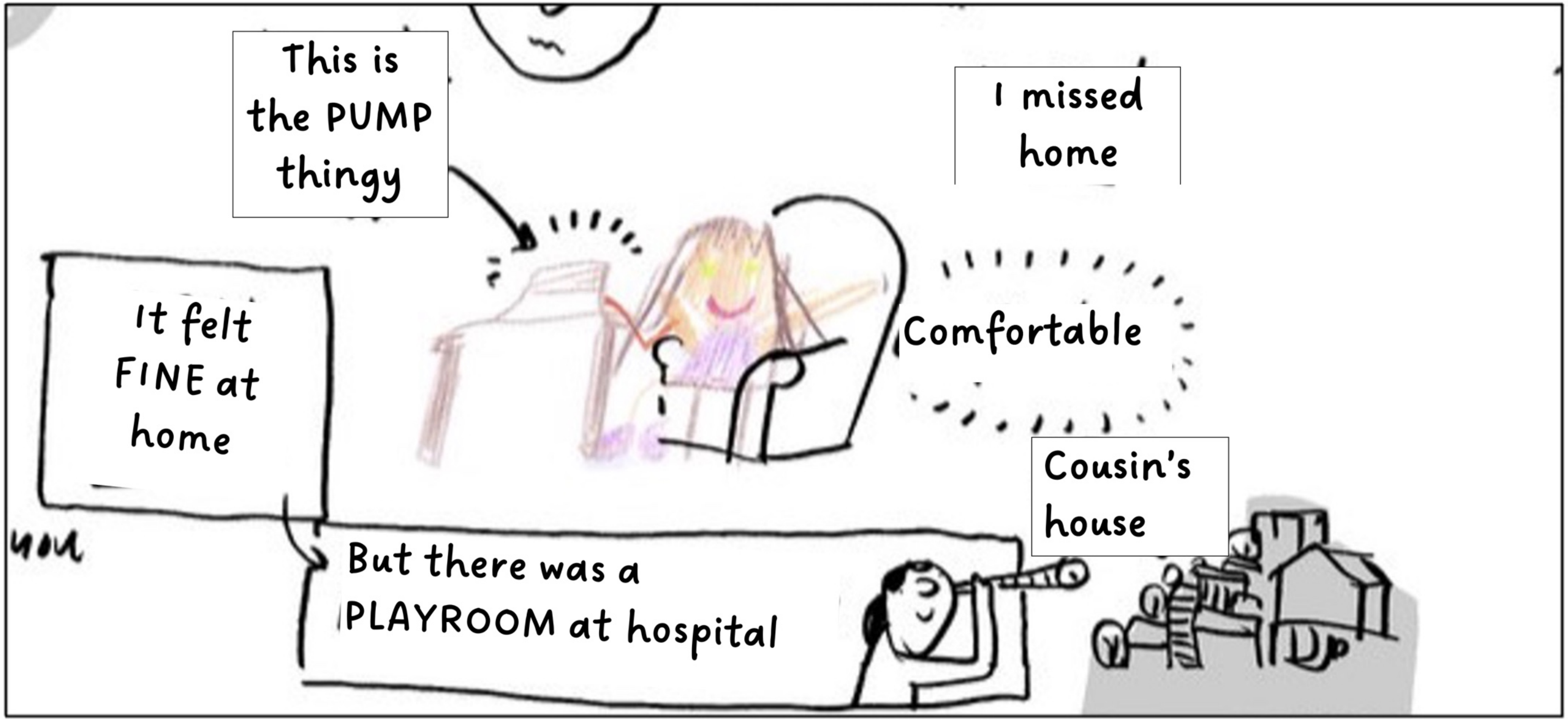

Figure S1. Child's drawing integrated into the illustration developed during the consultation activity (handwritten text changed to typed text for ease of reading).

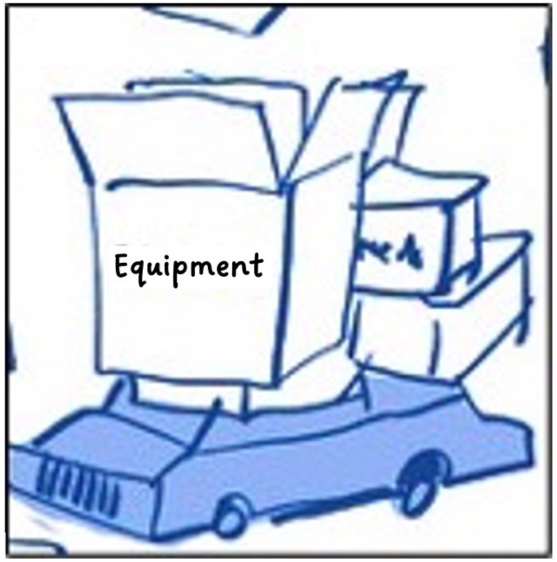

Figure S2. Example of an illustration created from a parent's account (handwritten text changed to typed text for ease of reading).

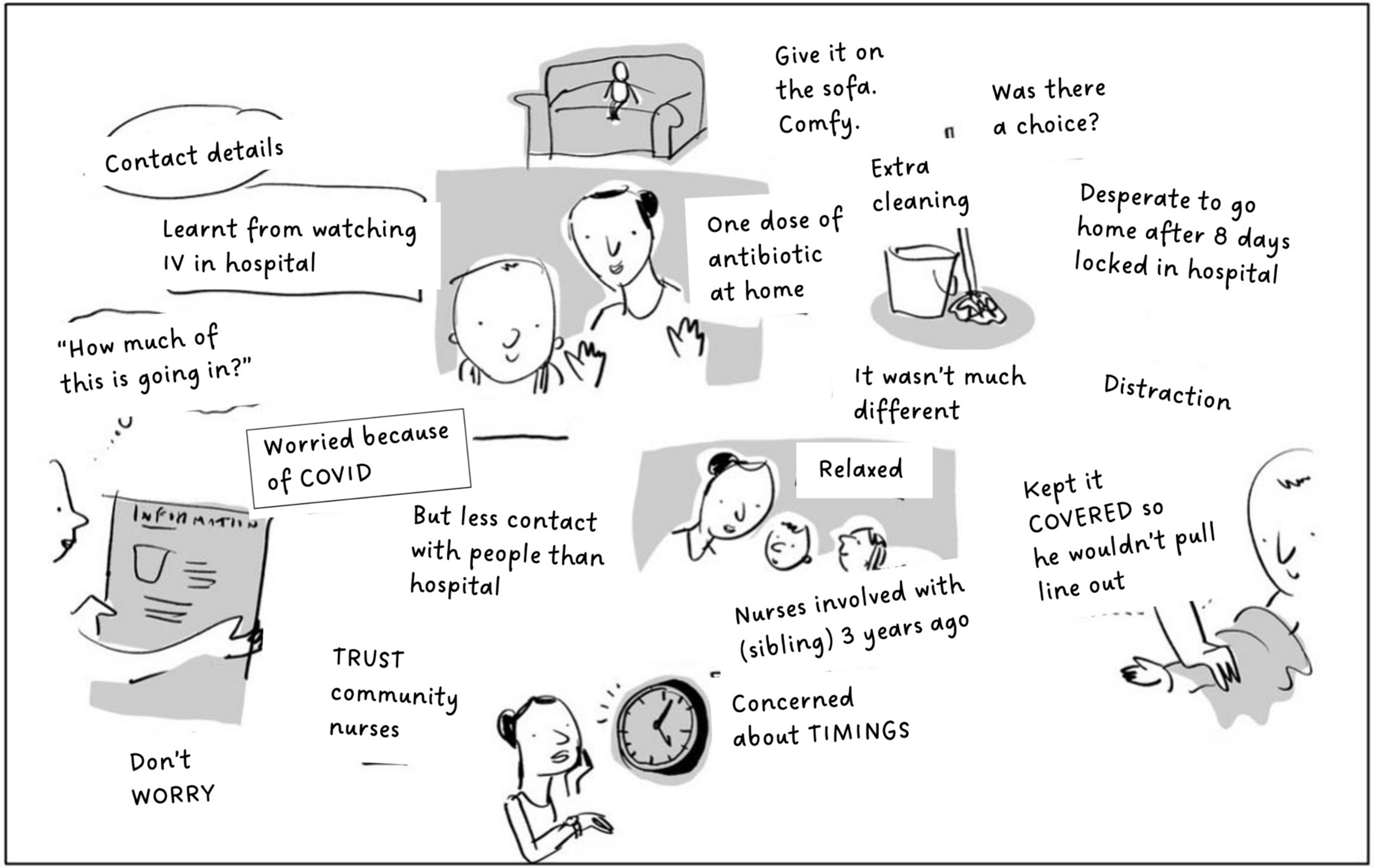

Figure S3. Annotated and illustrated 'map' of a child's experience [child's name redacted].
